# Hybrid Nanomodification of a Polymeric Asphalt Binder with Multiwalled Carbon Nanotubes and Nanoalumina to Enhance Microwave-Induced Healing and Asphalt Mixture Performance

**DOI:** 10.3390/nano16140882

**Published:** 2026-07-17

**Authors:** Luís Henrique Bissi Vidotti, João Victor Staub de Melo, Jaqueline Wolfart, Rafael Cassimiro Barbosa, Alexandre Luiz Manfro, Breno Salgado Barra, Carlos Eduardo Maduro de Campos

**Affiliations:** 1 Department of Civil Engineering, Federal University of Santa Catarina, Rua João Pio Duarte Silva, Florianópolis 88040-970, SC, Brazil; luis.vidotti@posgrad.ufsc.br (L.H.B.V.); jaqueline.wolfart@posgrad.ufsc.br (J.W.); rafael.cb@posgrad.ufsc.br (R.C.B.); alexandre.manfro@posgrad.ufsc.br (A.L.M.); breno.barra@ufsc.br (B.S.B.); 2 Department of Physics, Federal University of Santa Catarina, Rua Roberto Sampaio Gonzaga, Florianópolis 88035-972, SC, Brazil; carlos.campos@ufsc.br

**Keywords:** carbon nanotube, nanoalumina, asphalt mixture, fatigue damage, healing

## Abstract

Multiwalled carbon nanotubes (MWCNTs) and nanoalumina (nano-Al_2_O_3_) have each been studied separately in asphalt binders, but whether their combined thermal gain translates into microwave-induced healing at the mixture scale remains unestablished. This study aimed to evaluate, through a multiscale approach, their combined incorporation into a polymeric asphalt binder modified with 4% styrene-butadiene-styrene (SBS), focusing on mechanical performance and microwave-induced healing. Binders with 0 to 6% hybrid nanomaterial (50:50) were characterized structurally, chemically, rheologically, and thermally, and mixtures were evaluated for rutting, four-point bending fatigue, and microwave heating and healing. A content of 2.3% was selected from rheological and thermal criteria. At this content, the mixture heating rate rose from 0.18 to 0.41 °C/s (127.8%) and rut depth decreased by 22.1%. The nanomodified binder reduced the top-to-bottom Jnr_3.2_ gradient from over 250% to 56–59%, indicating improved storage compatibility rather than complete stability. Fatigue life at 250 μm/m decreased by 53.7%. Despite this, healing increased by 9.6% in dynamic modulus recovery and 61.9% in fatigue healing index. Overall, hybrid nanomodification improved resistance to permanent deformation and microwave-induced healing, clarifying their combined effect, although the fatigue penalty requires further investigation.

## 1. Introduction

Asphalt pavements are continuously subjected to repeated traffic loading, which induces cyclic stresses within the asphalt layers. Over time, repeated loading causes progressive material degradation, characterized by stiffness loss and the evolution of dispersed microcracks within the asphalt matrix into coalesced macrocracks. This process ultimately compromises pavement structural integrity and service life [1,2,3].

Within this context, the self-healing capability of asphalt materials has been widely investigated as a promising means of delaying damage evolution. This phenomenon relies on the intrinsic ability of the asphalt binder to partially restore material integrity during rest periods [4,5,6,7]. Healing is fundamentally associated with molecular diffusion and viscoelastic recovery and is strongly influenced by temperature, the mobility of the chemical fractions, and the rheological state of the binder [5,8,9].

However, polymer-modified binders, including those containing styrene-butadiene-styrene (SBS) copolymer, may exhibit a lower self-healing capability than conventional binders. Qiu et al. [10] demonstrated that SBS-modified asphalt mastic exhibited lower efficiency during both crack closure and mechanical strength recovery than a conventional mastic produced with a 70/100 penetration-grade binder. This behavior has been associated with the formation of a continuous polymer network within the binder, which alters its internal organization and viscoelastic response [11]. By restricting molecular mobility, this network may limit the molecular diffusion required for microcrack closure.

Strategies involving the application of external thermal stimuli have been proposed to enhance healing in both conventional and polymer-modified asphalt binders by increasing binder temperature and, consequently, fluidity [12,13,14]. Within this context, the incorporation of nanomaterials with high thermal conductivity represents a promising approach for improving heat transfer and temperature distribution within the asphalt matrix. These effects may enhance thermal response and healing efficiency, although they may also affect the mechanical performance of the material [13,14].

Recent studies have highlighted the use of metal oxide nanoparticles, such as ZnO, TiO_2_, and Al_2_O_3_, which may improve storage stability, healing capability, and binder rheology [15,16,17,18]. In particular, Broering et al. [16] reported that nanoalumina increased the thermal conductivity of an SBS-modified asphalt binder, thereby enhancing heat transfer within the material.

Carbon-based nanomaterials, such as multiwalled carbon nanotubes (MWCNTs), exhibit high thermal conductivity and can form interconnected conductive networks within the asphalt matrix. These networks may improve heat redistribution and intensify thermally activated healing processes [19,20]. However, the incorporation of carbon nanotubes alone may present limitations related to particle dispersion and excessive binder stiffening, particularly at high contents. These effects may adversely affect binder rheology and fatigue tolerance [21].

Combining MWCNTs and nanoalumina (nano-Al_2_O_3_) provides a hybrid approach involving nanomaterials from distinct material classes and with different morphologies: a one-dimensional carbon nanostructure and a particulate metal oxide. The high thermal conductivity of MWCNTs may enhance heat transfer, whereas nano-Al_2_O_3_ has been associated with improved thermal conductivity and storage compatibility in SBS-modified binders [22,23,24]. Previous studies by this research group investigated MWCNTs in conventional asphalt systems, focusing on mechanical properties such as rutting and fatigue resistance without evaluating induced healing [25]. MWCNTs were subsequently combined with steel wool to enhance microwave-induced healing [13]. Using the same aggregates, gradation, reference binder, and design binder content as those adopted in the present study, the research group also reported enhanced healing in SBS-modified asphalt mixtures containing 2.64 wt.% graphene nanoplatelets [14] or an 8.5 wt.% ZnO + TiO_2_ hybrid nanomaterial [18]. Nano-Al_2_O_3_ has also been shown to improve phase stability, although that investigation was limited to the binder scale [22]. Nevertheless, no previous study has established the combined effects of an MWCNT/nano-Al_2_O_3_ hybrid in an SBS-modified asphalt system, particularly regarding the balance among storage compatibility, microwave-induced heating and healing, rutting resistance, and fatigue performance.

Accordingly, this study evaluated an MWCNT/nano-Al_2_O_3_ hybrid in an SBS-modified asphalt system at both the binder and mixture scales. At the selected content of 2.3 wt.%, the hybrid enhanced microwave-induced heating and healing, improved resistance to permanent deformation, partially mitigated storage incompatibility, and reduced fatigue resistance. These findings identify the material-specific benefits and limitations of this hybrid nanomodification within an experimental framework previously established by the research group.

## 2. Materials and Methods

**Figure 1:**
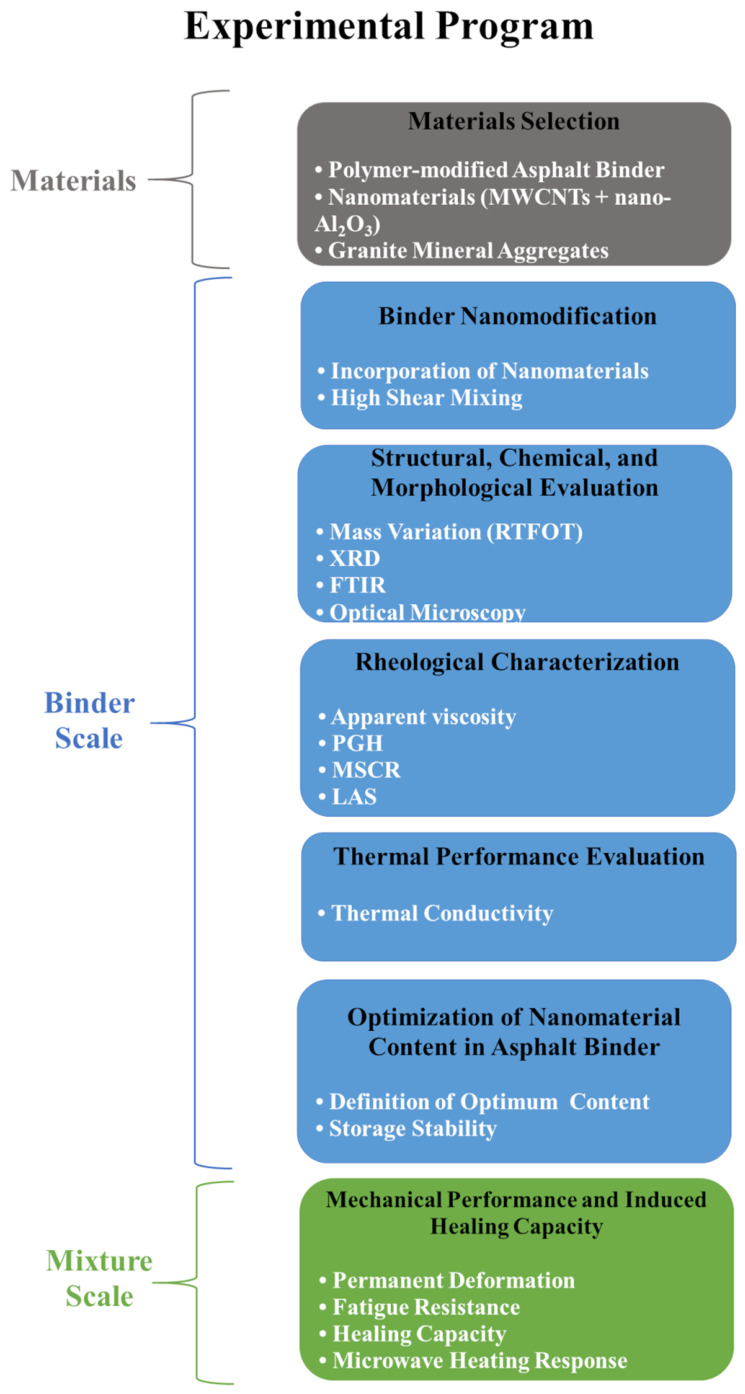
Experimental program comprising material selection, nanomodification of the reference binder, and performance evaluation at multiple scales.

This study adopted a multiscale approach to relate nanoscale modification of the SBS-modified asphalt binder to the macroscopic mechanical performance of the asphalt mixture. The experimental program was structured into three interdependent stages: (i) material selection and characterization; (ii) rheological and thermal characterization and selection of the nanomaterial content at the binder scale; and (iii) evaluation of the mechanical and healing properties at the mixture scale. The experimental workflow and sequence of tests are shown in Figure 1.

### 2.1. Materials

In this study, an asphalt binder industrially modified with 4 wt.% styrene-butadiene-styrene (SBS) copolymer was used as the reference binder and as the base binder for nanocomposite production. The physical properties of the binder are presented in Table 1.

Two nanomaterials were used to produce the asphalt nanocomposites: multiwalled carbon nanotubes (MWCNTs) and aluminum oxide nanoparticles (nano-Al_2_O_3_). MWCNTs were selected because of their high thermal conductivity and ability to form conductive networks within the asphalt matrix, potentially enhancing thermally activated healing mechanisms [19,20]. Nano-Al_2_O_3_ was selected because of its potential to increase thermal conductivity and improve binder storage stability and rheological behavior, as reported in the literature [16,22,23,24]. The physical and morphological properties of the nanomaterials are presented in Table 2.

Granitic aggregates supplied by the SBM quarry operated by Sul Brasil de Mineração in Paulo Lopes, Santa Catarina, Brazil, were used to produce the asphalt mixtures. A dense-graded asphalt mixture with a nominal maximum aggregate size of 19 mm was designed according to the formulation proposed by Melo [34], and the Superpave methodology [35], as applied before the implementation of Superpave 5. The aggregate gradation was selected to satisfy the applicable control limits. The physical and mechanical properties of the aggregates are presented in Table 3, and the selected gradation is presented in Table 4.

### 2.2. Preparation Protocol of Asphalt Nanocomposite

The nanomodification protocol began by manually premixing the MWCNTs and nano-Al_2_O_3_, which had previously been weighed at the specified ratio, to form the hybrid nanomaterial. The hybrid nanomaterial was then gradually incorporated into approximately 200 g of SBS-modified asphalt binder preheated to 160 ± 5 °C, corresponding to an apparent viscosity of 0.518–0.782 Pa·s. The material was initially stirred manually with a metal rod for 13 ± 2 min to promote preliminary dispersion and thermal equilibration before high-shear mixing. The system was subsequently mixed using a mechanical mixer (Mixer 700 Philco^®^, 700 W, Britânia Eletrodomésticos Ltda., Joinville, SC, Brazil) operating at 6000 rpm, while the binder temperature was maintained constant throughout the process. The mixing procedure comprised 20 consecutive cycles, each consisting of 1 min of mechanical mixing followed by 1 min of rest, for a total processing time of 40 min. This procedure was based on protocols previously used to incorporate nanomaterials into asphalt binders [47,48,49,50].

**Figure 2:**
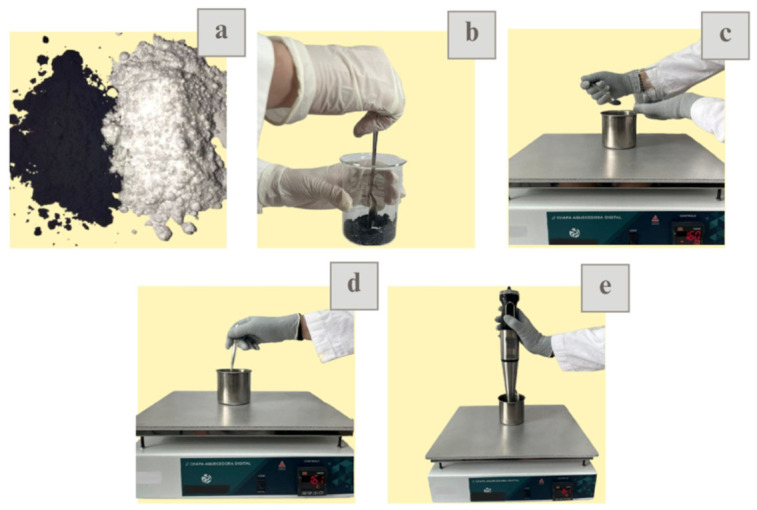
Preparation of the asphalt nanocomposite: (**a**) MWCNTs and nano-Al_2_O_3_, from left to right; (**b**) manual premixing of MWCNTs and nano-Al_2_O_3_; (**c**) manual incorporation of the hybrid nanomaterial into the asphalt binder at 160 ± 5 °C; (**d**) manual stirring of the hybrid nanomaterial and SBS-modified asphalt binder using a metal rod; and (**e**) high-shear mixing using a mechanical mixer.

Figure 2 illustrates the preparation of the asphalt nanocomposite.

The formulations evaluated in this study are presented in Table 5. Nanomaterial contents are expressed as percentages of the asphalt binder mass (wt.%).

A 50:50 MWCNT: nano-Al_2_O_3_ mass ratio was selected based on previous studies investigating the thermophysical behavior of hybrid systems containing carbon nanotubes and aluminum oxide nanoparticles [51,52]. Devarajan et al. [51] observed that the uniform dispersion of these nanoparticles increased the thermal conductivity of a hybrid nanofluid beyond that of suspensions containing only carbon nanotubes. A similar finding was reported by Hemmat Esfe et al. [52], who demonstrated that combining nanoparticles with different morphologies, such as carbon nanotubes and nano-Al_2_O_3_ particles, can produce a synergistic effect on the thermal conductivity of the system. The magnitude of this effect also depends on nanoparticle concentration. Because thermal conductivity is relevant to the performance of nanomodified asphalt binders, particularly during heating-induced healing, it was hypothesized that a similar synergistic effect could occur when the hybrid nanomaterial was incorporated into the asphalt matrix. Therefore, the 50:50 mass ratio was adopted as the initial formulation for investigating the effects of the MWCNT/nano-Al_2_O_3_ hybrid on the thermomechanical properties, storage stability, and healing performance of the asphalt matrix.

### 2.3. Structural, Chemical, and Morphological Evaluation of Asphalt Nanocomposite

After preparation, the asphalt nanocomposites were structurally, chemically, and morphologically characterized to investigate possible changes in the system before rheological testing. The samples were initially subjected to short-term aging according to ASTM D2872 [53], using a James Cox and Sons CS 325-B rolling thin-film oven (Colfax, CA, USA). Mass change was calculated as the percentage difference between the sample masses before and after aging. The 1% limit specified for asphalt binders in ASTM D6373 [54] was used as the reference criterion.

The structural organization of the nanocomposites was investigated by X-ray diffraction (XRD) using an X’Pert PRO Multi-Purpose diffractometer (Malvern Panalytical, Almelo, The Netherlands) with Cu Kα radiation. Scans were conducted over a 2θ range of 3–96°, using a step size of 0.100° and a counting time of 150 s. Three scans were performed for each sample. Unaged samples were molded into films approximately 1 mm thick and 25 mm in diameter.

Chemical compatibility and possible functional changes resulting from nanomodification were evaluated by Fourier-transform infrared spectroscopy (FTIR) using a Bruker VERTEX 70 spectrometer (Billerica, MA, USA). Spectra were collected over the range of 400–4000 cm^−1^ at a resolution of 4 cm^−1^, and 16 scans were averaged for each unaged sample.

The distribution of the nanomaterials within the asphalt matrix was qualitatively evaluated by bright-field optical microscopy using an Olympus BX41 microscope (Olympus Corporation, Tokyo, Japan) at 40× magnification. Thin films were deposited on glass slides and covered with coverslips.

This suite of analyses was used to assess the adequacy of the nanomodification protocol and identify evidence of degradation, incompatibility, or agglomeration that could adversely affect the subsequent rheological performance of the nanocomposites.

### 2.4. Rheological Characterization of Asphalt Binder

The rheological properties of the binders were characterized by rotational viscometry and dynamic shear rheometer (DSR) testing over a range of temperatures, aging conditions, and strain amplitudes.

The apparent viscosity of the unaged samples was measured using a Brookfield rotational viscometer (Middleboro, MA, USA) in accordance with ASTM D4402 [55]. Measurements were performed at 135, 150, and 177 °C.

Rheological tests were performed using a Discovery HR-2 dynamic shear rheometer (TA Instruments, New Castle, DE, USA). A 25 mm parallel-plate geometry with a 1 mm gap was used for high-temperature testing, whereas an 8 mm parallel-plate geometry with a 2 mm gap was used for intermediate-temperature testing at 20 °C.

The continuous high-temperature performance grade (PGH) was determined in accordance with ASTM D6373 [54] using the G*/sin δ parameter measured according to ASTM D7175 [56] for both unaged and RTFOT-aged samples. The Aging Index (AI) was calculated as the ratio of |G*|/sin δ after RTFOT aging to the corresponding value in the unaged condition.

The response of the binders to repeated loading at high temperature was evaluated using the Multiple Stress Creep Recovery (MSCR) test in accordance with ASTM D7405 [57]. The analysis focused on nonrecoverable creep compliance at 3.2 kPa (Jnr_3.2_) and percent recovery at 3.2 kPa (%R_3.2_), measured at the failure temperature of each asphalt nanocomposite.

Binder performance at intermediate temperature was evaluated using the Linear Amplitude Sweep (LAS) test at 20 °C in accordance with AASHTO T 391-20 [58]. The results were interpreted using the Simplified Viscoelastic Continuum Damage (S-VECD) model and the “AASHTO T 391-20—Version 1.59” spreadsheet [59]. Binder fatigue life was fitted using the power-law model presented in Equation (1).

(1) $$N_{f}=A\;{\left(\gamma \right)}^{B}$$

where A and B are the fatigue-curve coefficients, γ is the applied shear strain (%), and N_f_ is the number of cycles required to reach the failure criterion, defined as a 35% reduction in |G*|·sin δ.

The Binder Fatigue Factor (FFL) was calculated from the ratio of the fatigue lives obtained at strain amplitudes of 1.25% and 2.50%, as defined in Equation (2) [60]:

(2) $$\mathrm{FFL}\;=\;\frac{\log(N_{f\;(1.25)}\times N_{f\;(2.5)})}{2}\;\times \;\log\left(\frac{0.0250}{0.0125}\right)$$

where N_f(1.25)_ and N_f(2.5)_ denote the numbers of cycles to failure at strain amplitudes of 1.25% and 2.50%, respectively.

Table 6 summarizes the test procedures, aging conditions, and number of samples analyzed in each test.

### 2.5. Evaluation of Thermal Behavior of Binder

The thermal conductivity of the binders was measured using a C-Therm TCi Thermal Conductivity Analyzer (C-Therm Technologies Ltd., Fredericton, NB, Canada) equipped with a Modified Transient Plane Source (MTPS) sensor. RTFOT-aged specimens from nanocomposites S0–S6 were molded to a diameter of 25 mm and a thickness of 1 mm.

**Figure 3:**
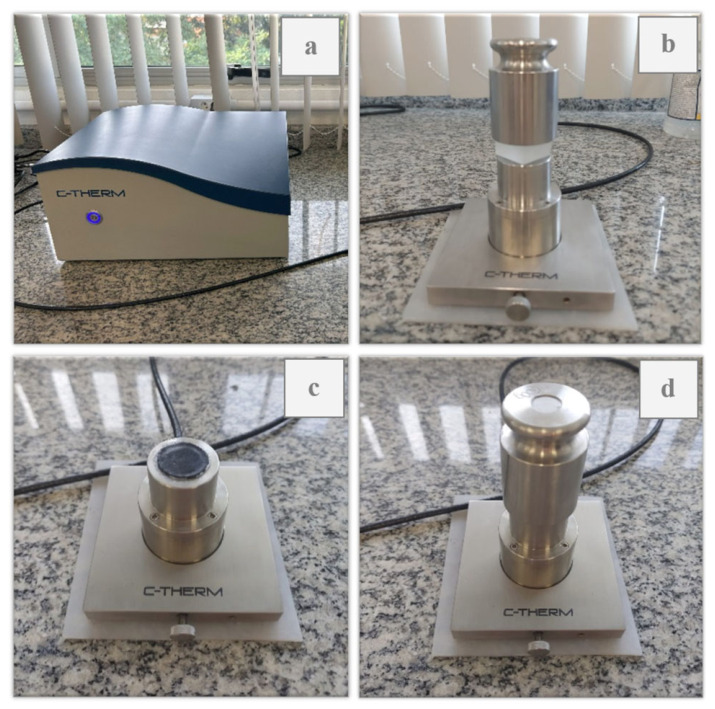
Procedure used to determine thermal conductivity: (**a**) C-Therm TCi equipment; (**b**) calibration using a reference material with a known thermal conductivity; (**c**) application of the thermal interface material and positioning of the asphalt binder specimen; and (**d**) specimen subjected to a constant load during data acquisition.

The equipment was calibrated using a reference material with a known thermal conductivity. To ensure adequate thermal contact between the specimen and sensor, a thin layer of thermal grease was applied before the specimen was positioned under a constant load during data acquisition, following the procedures described by Spínola et al. [14] and Wolfart et al. [18]. One specimen from each binder was evaluated, and six consecutive measurements were performed. The mean of these measurements was used as the final result. These repeated measurements characterize within-specimen repeatability rather than specimen-to-specimen variability. The experimental procedure is shown in Figure 3.

### 2.6. Definition of Nanomaterial Content in Polymer-Modified Asphalt Binder

#### 2.6.1. Selection of Nanomaterial Content

The nanomaterial content was selected based on an integrated assessment of rheological and thermal criteria, including thermal conductivity, continuous high-temperature performance grade (PGH), Aging Index, the MSCR parameters Jnr_3.2_ and %R_3.2_, and apparent viscosity. A maximum viscosity of 3000 cP at 135 °C was adopted as the workability limit in accordance with the Superpave methodology [62]. The application of these criteria and selection of the final content are described in Section 3.4.

#### 2.6.2. Evaluation of Storage Stability

After the nanomaterial content had been selected, storage stability was evaluated to assess the compatibility of the nanomodified system and its susceptibility to phase separation during high-temperature storage.

The test was conducted in accordance with ASTM D7173 [63] using unaged samples of the reference SBS-modified binder and the nanocomposite containing the selected nanomaterial content. Portions of 50 ± 0.5 g were placed in sealed aluminum tubes, held vertically at 163 ± 5 °C for 48 ± 1 h, and subsequently subjected to controlled cooling.

After conditioning, each tube was sectioned, and the top and bottom portions were subjected to MSCR testing [61] at the respective failure temperature of each binder. Comparison of the two portions enabled the assessment of rheological differences associated with phase separation and, consequently, the storage compatibility of the nanomodified binder before its use in asphalt mixture production.

### 2.7. Evaluation of Mechanical Performance and Healing Capacity of Asphalt Mixture

The asphalt mixture design was based on the optimum binder content reported by Manfro [48], because the same mineral aggregates, aggregate gradation, and reference SBS-modified binder were used. The adopted design binder content was 4.44%, as determined from the Superpave volumetric criteria for heavy traffic [35,64,65].

Two asphalt mixtures were evaluated: (i) a reference mixture (Mr), produced with the asphalt binder containing 4 wt.% SBS; and (ii) a nanomodified mixture (Mn), produced with the nanocomposite containing the nanomaterial content selected in Section 2.6.1. Both mixtures were produced at the design binder content of 4.44%. Their performance was evaluated in terms of permanent deformation, dynamic modulus and phase angle, fatigue resistance, healing capacity, and microwave-heating response. Table 7 summarizes the experimental program, including the number and dimensions of the specimens, equipment, and applicable standards or procedures.

In four-point bending fatigue test [67], failure was defined as a 50% reduction in the initial dynamic modulus measured at the 100th loading cycle. Fatigue life (N_f_) was fitted using the power-law model presented in Equation (3).

(3) $$N_{f}=a\;{\left(\varepsilon \right)}^{-b}$$

where *ε* is the applied strain (μm/m), and a and b are coefficients obtained by logarithmic regression.

The Mixture Fatigue Factor (FFM) was also calculated according to Equation (4) [68].

(4) $$\mathrm{FFM}=0.2\left[\log(N_{100})+\log(N_{250})\right]$$

where N_100_ and N_250_ denote the numbers of cycles to failure at strain amplitudes of 100 and 250 μm/m, respectively, using base-10 logarithms.

After each fatigue test was interrupted at the failure criterion, the specimen was subjected to the microwave-healing protocol for 140 s using an Electrolux ME28S microwave oven operating at 900 W and 2450 MHz (Manaus, Amazonas, Brazil). The specimen was subsequently allowed to rest for 3 h at 20 °C and was then retested under the same initial test conditions. This procedure followed methodologies previously applied in related studies [36,49,50], thereby enabling comparisons among the results.

Healing capacity was evaluated by comparing material performance before and after microwave heating in terms of the number of cycles to failure and recovery of the initial dynamic modulus.

Fatigue Healing Index (FHI) was determined according to Equation (5).

(5) $$\mathrm{FHI}=\frac{N_{\mathrm{healed}}}{N_{\mathrm{original}}}$$

where N_original_ is the number of cycles to failure during the first fatigue stage, and N_healed_ is the number of cycles to failure after the healing protocol.

Dynamic Modulus Healing Index (DMHI) was calculated according to Equation (6).

(6) $$\mathrm{DMHI}=\frac{|E^{*}{|}_{\mathrm{initial},\mathrm{healed}}}{|E^{*}{|}_{\mathrm{initial},\mathrm{original}}}$$

where |E*|_initial,original_ and |E*|_initial,healed_ are the dynamic modulus values measured at the 100th cycle before and after the healing protocol, respectively.

The healing performance of each mixture was also normalized by the energy density associated with microwave heating. The result for the nanomodified mixture was compared with that of the reference mixture reported by Wolfart et al. [18].

**Figure 4:**
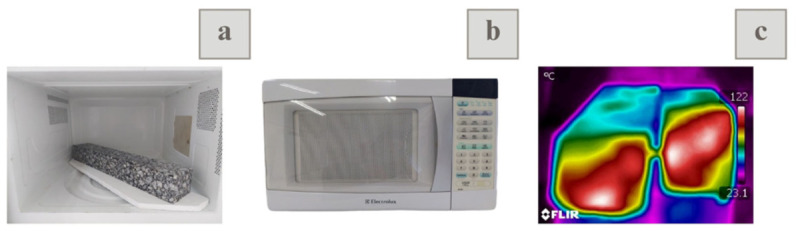
Determination of internal temperature after microwave heating: (**a**) nanomodified asphalt mixture specimen during heating; (**b**) microwave oven; and (**c**) thermographic image of the cross-section obtained immediately after sectioning, showing the temperature distribution after 183 s of heating.

After microwave heating, the mean internal temperature of the asphalt mixtures was determined at different heating durations. The temperature of the nanomodified mixture was measured experimentally in this study, whereas the corresponding values for the reference mixture were obtained from Wolfart et al. [18]. Immediately after heating, each specimen was transversely sectioned, and the freshly exposed cross-sectional surface was examined using a FLIR B400 infrared thermal camera (Wilsonville, OR, USA). Figure 4 shows a representative example of the procedure, including microwave heating and the thermographic image obtained after sectioning. The mean internal temperature was estimated as the arithmetic mean of the maximum and minimum temperatures recorded across the analyzed section, based on the thermal scale provided by the equipment. This procedure provided a representative estimate of the internal temperature immediately after heating.

## 3. Results and Discussion

### 3.1. Structural, Chemical, and Morphological Evaluation of Asphalt Nanocomposite

The nanocomposites were characterized before rheological testing to evaluate nanomaterial incorporation and identify possible changes in the system that could affect rheological performance.

#### 3.1.1. Mass Change After Short-Term Aging (RTFOT)

The mass-change results after short-term aging in the RTFOT are presented in Table 8. All asphalt nanocomposites exhibited mass changes below 0.40%, which is lower than the maximum limit of 1% specified in ASTM D6373 [54].

The combined incorporation of MWCNTs and nano-Al_2_O_3_ did not produce an appreciable increase in volatilization or loss of the binder’s light fractions during short-term aging. Nanocomposites S1 and S2 exhibited slightly lower values than the reference binder (0.31% versus 0.34%), whereas S3, S4, and S6 exhibited slightly higher values (0.39–0.40%). Nevertheless, all results remained within a narrow and technically comparable range.

These results suggest that the presence of the nanomaterials did not compromise the thermal stability of the system. The low standard deviations (≤0.01%) indicate good measurement repeatability and consistency in the nanomodification process. Overall, the incorporation of MWCNTs and nano-Al_2_O_3_ did not appreciably affect susceptibility to mass loss during aging, and all nanomodified binders remained within the specification limit.

#### 3.1.2. X-Ray Diffraction (XRD)

**Figure 5:**
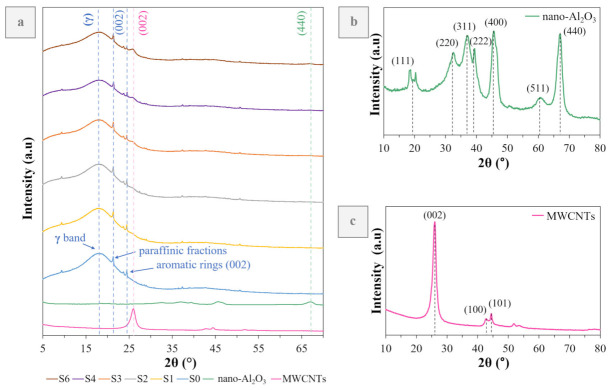
X-ray diffraction pattern: (**a**) nanomaterial, reference binder (S0), and nanocomposite (S1–S6), unaged condition; (**b**) planes of nano-Al_2_O_3_; (**c**) planes of MWCNTs.

X-ray diffraction analysis was conducted to characterize the crystalline phases of the nanomaterials and investigate possible structural changes in the asphalt binder resulting from the incorporation of MWCNTs and nano-Al_2_O_3_. The diffraction patterns of the reference binder, nanocomposites, and individual nanomaterials are presented in Figure 5.

The MWCNT diffraction pattern exhibited its most intense peak at 2θ = 26°, which was attributed to the (002) plane and is characteristic of stacked graphitic layers. Reflections associated with the (100) and (101) planes were observed at 2θ = 43–44° and were related to the lateral ordering of the graphitic structure. This pattern is characteristic of multiwalled carbon nanotubes and is consistent with previous reports [69,70,71].

For nano-Al_2_O_3_, reflections were identified at approximately 18.5°, 19.6°, 20.3°, 32.5°, 37.0°, 39.2°, 45.6°, 60.6°, and 67° (2θ). Except for the peaks at 18.5° and 20.3°, these reflections were assigned to the (111), (220), (311), (222), (400), (511), and (440) planes, respectively. They are consistent with the cubic spinel structures of γ-Al_2_O_3_ (JCPDS 01-079-1558) and η-Al_2_O_3_ (JCPDS 01-079-1557). Similar diffraction patterns have been reported for nano-Al_2_O_3_ with comparable structures [22,72,73]. The additional reflections, particularly those at 18.5° and 20.3°, could not be conclusively assigned to a distinct phase. Nevertheless, the pattern was predominantly characterized by the reflections expected for the crystalline phases of the nanomaterial.

The reference SBS-modified binder exhibited a predominantly amorphous pattern, characterized by a broad halo centered at 2θ = 18° (γ band), which is typical of bituminous materials containing saturated hydrocarbons. A signal at 2θ = 24° was attributed to the stacking of aromatic rings along the (002) plane. A discrete peak was also observed at 2θ = 21° and has frequently been associated with paraffinic fractions or partially ordered domains [74,75,76].

At the higher nanomaterial contents, particularly 6 wt.%, the nanocomposites exhibited reflections associated with the (002) plane of MWCNTs at 26° and the (440) plane of nano-Al_2_O_3_ at 67°. These reflections indicate the presence of the crystalline nanomaterial phases within the asphalt matrix. At lower contents, identification of these peaks was limited by the predominantly amorphous response of the binder, as expected for systems containing low reinforcement contents. No new peaks or shifts in angular position were observed, indicating that no new crystalline phases were detected after nanomodification.

#### 3.1.3. Fourier Transform Infrared Spectroscopy (FTIR)

**Figure 6:**
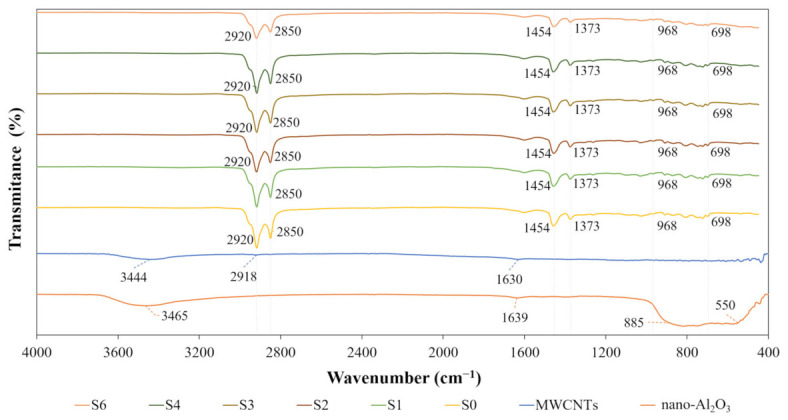
FTIR spectra of reference polymer-modified asphalt binder, nanocomposite containing different content of MWCNTs + nano-Al_2_O_3_, and nanomaterial.

Possible chemical changes resulting from the incorporation of the nanomaterials into the SBS-modified asphalt binder were investigated by Fourier-transform infrared spectroscopy (FTIR). Figure 6 presents the FTIR spectra of the reference binder, nanocomposites, and individual nanomaterials in the unaged condition.

The reference binder exhibited the characteristic bands of the SBS copolymer. The band at 699 cm^−1^ was attributed to the out-of-plane C-H bending vibration of aromatic rings in the polystyrene (PS) segments, whereas the band at 968 cm^−1^ corresponded to the out-of-plane vibration of the -CH=CH- bonds in the polybutadiene (PB) segments. These bands confirm the presence of the copolymer within the asphalt matrix [77,78]. The bands at 2920 and 2850 cm^−1^ were attributed to the C-H stretching vibrations of aliphatic CH_2_ and CH_3_ groups, whereas those at 1458 and 1373 cm^−1^ corresponded to the deformation of these groups in saturated hydrocarbons [18,79].

The MWCNT spectrum exhibited a band at 3444 cm^−1^ associated with surface O-H groups or adsorbed water, a peak at 2918 cm^−1^ associated with C-H bonds, and a band at 1630 cm^−1^ attributed to the graphitic C=C structure, consistent with previous reports for carbon nanotubes [80,81,82].

The nano-Al_2_O_3_ spectrum exhibited a broad band between approximately 550 and 885 cm^−1^, corresponding to Al-O and Al-O-Al vibrations. Bands at 3465 and 1639 cm^−1^ were associated with O-H stretching and H-O-H deformation, respectively, indicating the presence of surface hydroxyl groups and adsorbed water [22,83,84].

In the asphalt nanocomposites, the main characteristic bands of the asphalt matrix were preserved, and no new bands were observed. The spectral contribution of the nanomaterials could not be clearly distinguished, possibly because of their relatively low contents and the spectral dominance of the bituminous matrix. Preservation of the characteristic binder bands, together with the absence of relevant new bands, indicates that the incorporation of MWCNTs and nano-Al_2_O_3_ did not produce detectable chemical changes within the sensitivity range of the technique. Although this result does not establish a specific interaction mechanism, it is consistent with nanomodification predominantly governed by physical and interfacial interactions [22,85].

#### 3.1.4. Bright-Field Optical Microscopy

**Figure 7:**
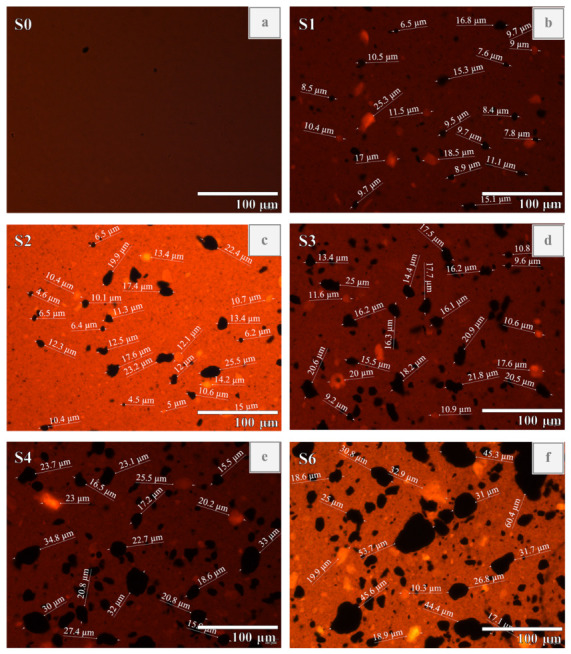
Bright-field optical micrographs (40×) of the SBS-modified asphalt binder containing different nanomaterial contents: (**a**) S0, reference binder; (**b**) S1; (**c**) S2; (**d**) S3; (**e**) S4; and (**f**) S6, all in the unaged condition.

Bright-field optical microscopy was used to qualitatively evaluate the morphology and distribution of the nanomaterials within the asphalt matrix. Representative micrographs are presented in Figure 7.

The reference binder (Figure 7a) exhibited a homogeneous microstructure, with no dark domains associated with particle incorporation. In the nanocomposites containing total nanomaterial contents of 1% and 2% (S1 and S2; Figure 7b,c), small dark domains were relatively uniformly distributed throughout the asphalt matrix. This observation indicates visually homogeneous nanomaterial dispersion without substantial agglomeration.

At a total nanomaterial content of 3% (Figure 7d), the density of these domains increased and the distance between particles appeared to decrease. At higher contents of 4% and 6% (Figure 7e,f), larger agglomerates and a less homogeneous distribution were observed, indicating an increasing tendency toward agglomeration. Overall, dispersion was more uniform at contents of up to 3%, whereas the binders containing 4% and 6% exhibited greater agglomeration. This behavior is commonly associated with stronger particle-particle interactions in systems containing high nanomaterial contents. In nanomodified systems, more homogeneous dispersion may promote distributed reinforcement throughout the matrix, whereas agglomeration may increase local stiffness, restrict flow, and reduce the efficiency of stress transfer [86,87].

#### 3.1.5. Integrated Analysis of Results

Taken together, the RTFOT, XRD, FTIR, and optical microscopy results indicate that nanomaterial incorporation occurred without evidence of thermal degradation or the formation of new detectable crystalline phases. Mass changes below the specification limit indicated low susceptibility to the loss of light fractions during short-term aging. The XRD patterns confirmed that the binder retained its predominantly amorphous character, although reflections attributable to the nanomaterials became detectable at higher contents. The FTIR spectra showed that the principal binder bands were preserved, with no relevant new bands or substantial shifts. Optical microscopy indicated more uniform dispersion at contents of up to 3% and a greater tendency toward agglomeration at 4% and 6%.

In the absence of evidence of chemical reactions, these findings suggest that the changes observed in rheological and mechanical performance, discussed in the following sections, may arise predominantly from the physical and microstructural effects of nanomaterial incorporation.

### 3.2. Rheological Characterization of Asphalt Binder

This section addresses the rheological behavior at the binder scale, before mixture production. The seven binders produced with MWCNTs + nano-Al_2_O_3_ contents ranging from 0% to 6% were evaluated for apparent viscosity (Section 3.2.1), high-temperature performance grade and aging susceptibility (Section 3.2.2), susceptibility to permanent deformation (Section 3.2.3), and fatigue damage tolerance (Section 3.2.4). Throughout, the reference binder is the sample without nanomaterial (S0), which appears in all figures as the 0% content and provides the basis for every percentage variation reported.

#### 3.2.1. Apparent Viscosity

**Figure 8:**
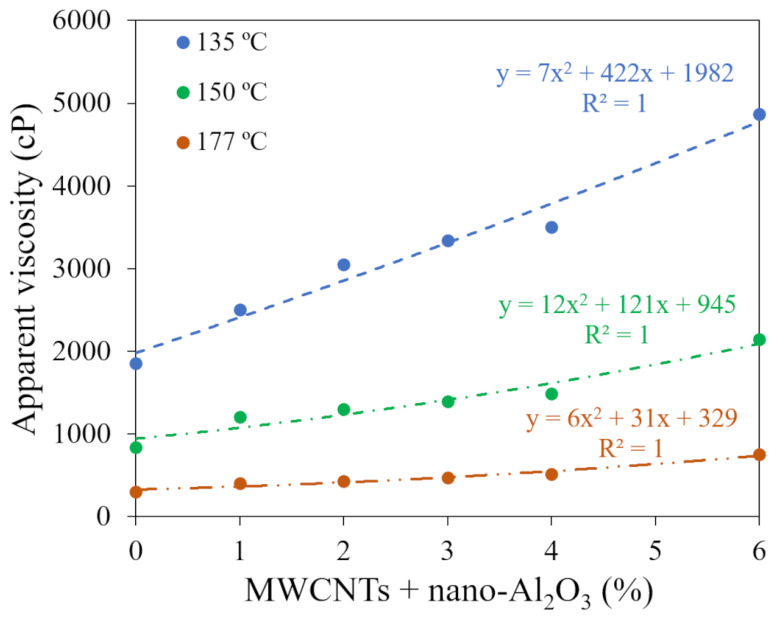
Apparent viscosity of binders as a function of MWCNTs + nano-Al_2_O_3_ content at 135 °C, 150 °C, and 177 °C.

Figure 8 presents the variation in binder apparent viscosity as a function of MWCNT + nano-Al_2_O_3_ content at 135, 150, and 177 °C.

A progressive increase in viscosity is observed in Figure 8 with increasing nanomaterial content at all evaluated temperatures. For 6% addition, the variations relative to the reference binder were 140.5% (135 °C), 122.5% (150 °C), and 122.2% (177 °C). The Superpave methodology [62] establishes a maximum limit of 3.0 Pa·s (3000 cP) at 135 °C to ensure adequate workability during mixing and pumping. The trendline indicates that the 2.3% content is at the limit of this criterion, while higher contents exceed the recommended range. A second-order polynomial was fitted to describe the nonlinear relationship between nanomaterial content and viscosity within the tested range. The fit was used only to identify the content satisfying the workability limit. In this context, for the 2.3% content, the increases corresponded to 50.8%, 36.2%, and 31.3% at 135 °C, 150 °C, and 177 °C, respectively.

This behavior is consistent with the microstructural evidence (Section 3.1.4), which indicates an increase in the size and connectivity of agglomerates, potentially resulting in greater resistance to flow. Similar results have been reported for nanomodified binders containing carbon nanotubes and nano-Al_2_O_3_ used individually [22,23,24,88,89,90].

The progressive increase in viscosity can be interpreted as a consequence of increased resistance to flow of the binder, promoted by the high specific surface area of the nanomaterials and the formation of more interconnected domains as the content increases. The elongated geometry of both nanomodifiers favors physical interactions that hinder flow. This interpretation is consistent with studies that report increased viscosity and higher rheological sensitivity with increasing content of CNTs or nano-Al_2_O_3_ in nanomodified binders [22,86,87].

#### 3.2.2. Determination of High-Temperature Performance Grade

**Figure 9:**
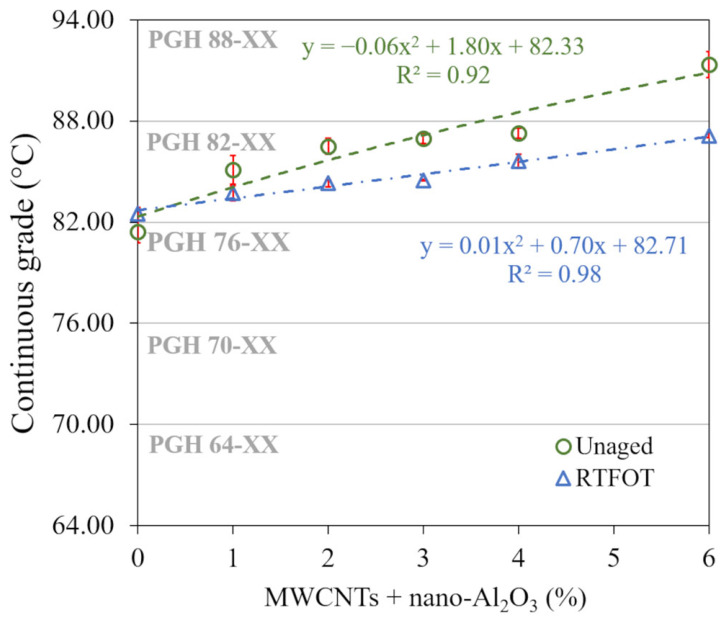
Comparison of high-temperature performance grade (PGH) of the reference binder and the nanocomposites (S0–S6) under unaged and short-term aged (RTFOT) conditions. Error bars: standard deviations.

Figure 9 presents the variation in high-temperature performance grade (PGH) of the asphalt nanocomposites under unaged and short-term aged (RTFOT) conditions as a function of MWCNTs + nano-Al_2_O_3_ content.

In general, the results indicate a progressive increase in the parameter with increasing nanomaterial content under both conditions. In the unaged state, PGH increased from 82.33 °C to 90.97 °C when the content increased from 0% to 6%, corresponding to a gain of 10.5%. For the binders subjected to RTFOT, the increase was from 82.71 °C to 87.27 °C, corresponding to 5.5%. These results indicate that nanomodification promotes increased resistance to permanent deformation, with a more pronounced effect before aging.

The trendline indicates that the transition from PGH 82-XX to PGH 88-XX occurred at approximately 3.6% combined addition, considering the unaged condition. Although no grade shift was observed for the aged binders (RTFOT), a consistent increase in the parameter with increasing nanomaterial content was observed. This behavior is consistent with previous studies that report increased stiffness at high-temperatures in nanomodified binders containing carbon nanotubes and nano-Al_2_O_3_ [16,86].

**Figure 10:**
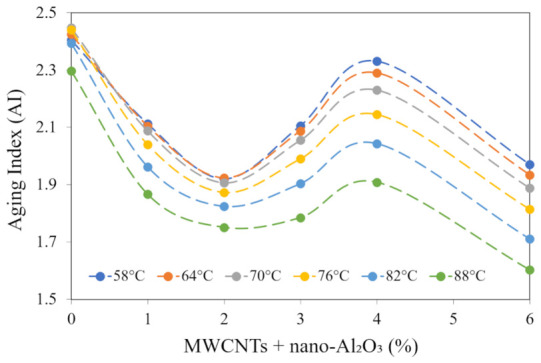
Aging Index (AI) as a function of nanomaterial content (MWCNTs + nano-Al_2_O_3_) at different test temperatures.

Resistance to aging was evaluated using the Aging Index (AI), calculated from the ratio |G*|/sin δ between the unaged and short-term aged conditions. The results are presented in Figure 10.

A reduction in AI indicates greater resistance to oxidation during short-term aging. A consistent decrease in the index is observed with the incorporation of MWCNTs + nano-Al_2_O_3_ up to 2%, with reductions of approximately 20.0% (58 °C) to 23.9% (88 °C) relative to the reference binder, depending on the test temperature. For contents between 3% and 4%, an increase in AI is observed, although the values remain lower than those of the reference, with reductions ranging from 2.9% to 22.6%. At 6%, a further reduction in the index is observed, reaching the lowest AI values, with reductions of up to 33.1% (88 °C), especially at higher temperatures. Overall, the results indicate that the incorporation of nanomaterials improves resistance to short-term aging, with more pronounced performance at higher contents.

#### 3.2.3. Multiple Stress Creep Recovery (MSCR)

**Figure 11:**
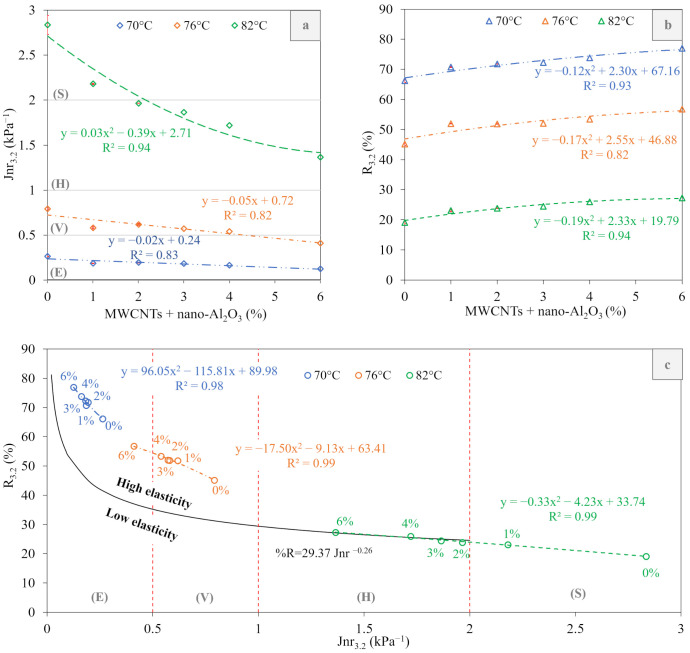
MSCR parameters of RTFOT-aged asphalt binders as a function of MWCNT + nano-Al_2_O_3_ content at 70, 76, and 82 °C: (**a**) nonrecoverable creep compliance at 3.2 kPa (Jnr_3.2_); (**b**) percent recovery at 3.2 kPa (%R_3.2_); and (**c**) elastic-response classification. Error bars represent standard deviations. The abbreviations represent the traffic level classifications: Standard (S), Heavy (H), Very Heavy (V), and Extremely Heavy (E).

Figure 11a presents the nonrecoverable creep compliance (Jnr_3.2_) values for the binders short-term aged (RTFOT), evaluated at 70, 76, and 82 °C. A progressive reduction in Jnr_3.2_ is observed with increasing MWCNTs + nano-Al_2_O_3_ content at all temperatures. Comparing the reference binder (0%) with the 6% content, the reduction in Jnr_3.2_ was 50.0% (70 °C), 41.7% (76 °C), and 46.5% (82 °C), indicating lower susceptibility to permanent deformation under a stress level of 3.2 kPa.

Regarding traffic level classification according to ASTM D8239 [57], no change was observed at 70 °C. However, at 76 °C, the fitted trend indicates that contents ≥ 4.2% promote a transition from Very Heavy (V) to Extremely Heavy (E). At 82 °C, contents ≥ 2.3% result in a transition from Standard (S) to Heavy (H).

Simultaneously, Figure 11b shows that the percent recovery at 3.2 kPa (%R_3.2_) increased with the incorporation of nanomaterials. For the 6% content, the increases relative to the reference binder were 14.1% (70 °C), 19.6% (76 °C), and 36.1% (82 °C). The %R_3.2_ versus Jnr_3.2_ diagram (Figure 11c) indicates that the experimental points shift toward the region of lower Jnr_3.2_ and higher %R_3.2_, maintaining the binder within the high elasticity region according to AASHTO R 92 [91] (70 °C and 76 °C), while exhibiting a response that is simultaneously more resistant to permanent deformation and more recoverable.

The reduction in Jnr_3.2_ together with the increase in %R_3.2_ indicates that the hybrid system not only increased binder stiffness but also enhanced the recoverable response under repeated loading. This behavior is consistent with a physical reinforcement of the matrix and with a greater restriction to irreversible strain of the continuous phase, although this mechanism was not directly observed in the present study. The literature on nano-Al_2_O_3_ and CNTs in asphalt binders also reports improvement in parameters associated with resistance to permanent deformation and elastic recovery under MSCR conditions [22,23,86,88].

#### 3.2.4. Fatigue Damage Tolerance

**Figure 12:**
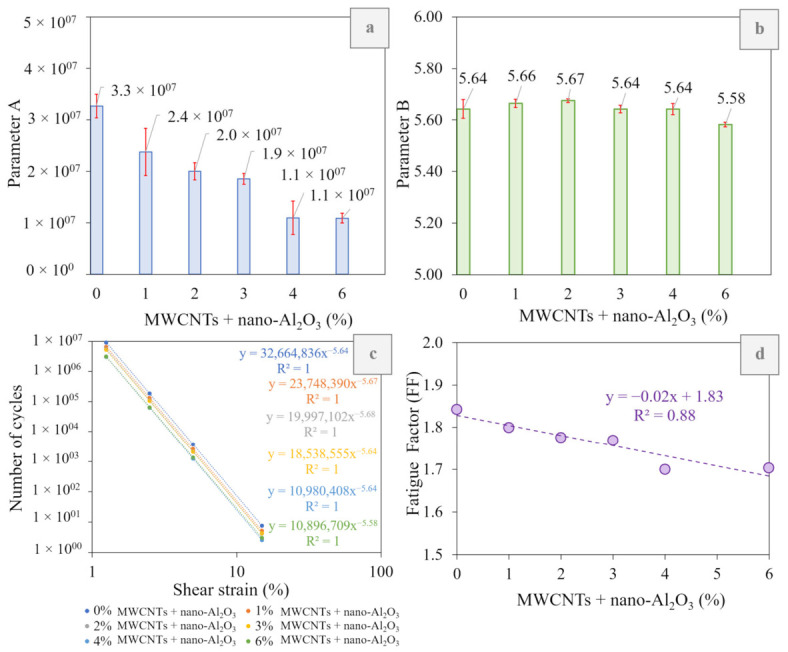
Fatigue response of asphalt nanocomposites (after short-term aging) evaluated by LAS test [58]: (**a**) fatigue parameter A; (**b**) fatigue parameter B; (**c**) fatigue curves; and (**d**) binder fatigue factor (FFL) derived from LAS analysis. Error bars: standard deviations.

Fatigue damage tolerance was evaluated using the Linear Amplitude Sweep (LAS) test, according to AASHTO T 391-20. Figure 12 presents the fatigue response of asphalt nanocomposites after short-term aging (RTFOT).

Parameter A (Figure 12a), associated with initial structural integrity of the binder, showed a progressive reduction with increasing nanomaterial content. Comparing the reference binder with the nanocomposites containing 4% and 6% MWCNTs + nano-Al_2_O_3_, the value of parameter A decreased by 66.7%, from 3.3 × 10^7^ to 1.1 × 10^7^. This behavior indicates a decrease in the initial damage resistance capacity under cyclic loading, consistent with the increase in binder stiffness at high-temperatures, as evidenced by the reduction in Jnr_3.2_ in the MSCR test and the increase in PGH.

Parameter B (Figure 12b), related to strain sensitivity, showed low variation with increasing nanomaterial content. A slight increase of 0.4% and 0.5% was observed at intermediate contents (1% and 2%) relative to the reference binder, followed by stabilization at 3% and 4%, and a slight reduction of 1.1% at 6% MWCNTs + nano-Al_2_O_3_. Overall, the variations remain within a narrow range (0.4–1.1%), indicating that the slope of the fatigue curve remains essentially unchanged. This behavior suggests that hybrid nanomodification predominantly affects the magnitude of fatigue life, without significantly altering strain sensitivity.

The reduction in parameter A (Figure 12a), combined with the small variation in B (Figure 12b), indicates that hybrid nanomodification affected initial structural integrity of the binder more significantly than its strain sensitivity. This finding suggests that the main effect of nanomaterial incorporation was to shift the fatigue curve (Figure 12c) toward lower fatigue life levels, without significantly altering how the response evolves with increasing strain.

The binder fatigue factor (FFL) (Figure 12d) showed a decreasing trend with increasing MWCNTs + nano-Al_2_O_3_ content, indicating a reduction in overall fatigue damage tolerance. This trend is consistent with the reduction observed in parameter A and with the shift in fatigue curves (Figure 12c), indicating a decrease in fatigue life of nanomodified binders.

### 3.3. Evaluation of Thermal Behavior of Binders

**Figure 13:**
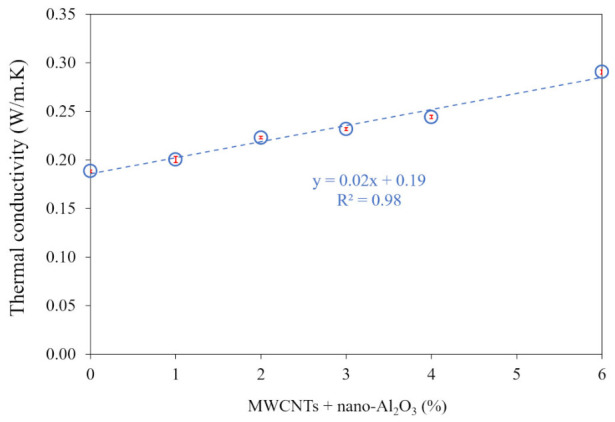
Thermal conductivity of asphalt binders aged by RTFOT as a function of nanomaterial content (MWCNTs + nano-Al_2_O_3_). Error bars: standard deviations.

The thermal conductivity of the RTFOT-aged asphalt nanocomposites was evaluated to determine the heat-conduction capacity of the nanomodified binders and its potential contribution to thermally induced healing. Measurements were conducted at a mean ambient temperature of 24.5 °C (standard deviation = 0.1 °C), and the results are presented in Figure 13.

A progressive increase in thermal conductivity is observed with increasing MWCNTs + nano-Al_2_O_3_ content, with a mean rate of approximately 0.02 W/m·K per percentage point of nanomaterial. Relative to the reference binder, the nanocomposite containing 6% exhibited an increase from 0.19 W/m·K to 0.31 W/m·K, corresponding to 63.2%. A similar trend was reported by Broering et al. [16], who observed an increase in thermal conduction in SBS nanomodified binders containing nano-Al_2_O_3_.

The progressive increase in thermal conductivity shows that the heat conduction capacity of the matrix increased with nanomaterial content. This behavior is consistent with the hypothesis that the hybrid phase established more efficient conduction paths within the matrix [20,22,51], although such paths were not directly observed. Regardless of the mechanism, the higher conductivity may promote greater binder mobility during microwave heating, potentially enhancing fatigue damage healing.

### 3.4. Definition of Nanomaterial Content in Polymer-Modified Asphalt Binder

Table 9 presents the rheological and thermal properties of the asphalt nanocomposites. Because thermal conductivity, PGH, and %R_3.2_ increased monotonically with nanomaterial content, whereas Jnr_3.2_ decreased, the final content was selected based primarily on the workability constraint.

Since thermal conductivity, PGH, Jnr_3.2_, and %R_3.2_ improve monotonically with content, the selection was governed by the workability constraint. The polynomial fit of the viscosity data indicates that 2.3% is the highest content within the 3.0 Pa·s limit at 135 °C, while the 3.0% content exceeded it. The binder produced at this content was characterized in the storage stability test (Section Storage Stability) and used in all asphalt mixtures (Section 3.5).

Relative to the reference binder, the selected content resulted in an increase of 50.8% in apparent viscosity at 135 °C, gains of 4.6% and 2.0% in PGH for unaged and RTFOT conditions, respectively, a mean increase of 13.2% in %R_3.2_, a mean reduction of 20.8% in Jnr_3.2_, a mean reduction of 21.3% in Aging Index between 58 and 88 °C, and an increase of 24.2% in thermal conductivity.

The results of the Linear Amplitude Sweep (LAS) test [58], although relevant for characterization of fatigue damage tolerance, were considered complementary in the selection of nanomaterial content. This choice is related to the phenomenological nature of the test and the still limited direct correspondence between fatigue response of the binder and that of the asphalt mixture [92]. In addition, although the binder fatigue factor decreased with increasing nanomaterial content, the 2.3% content represented an intermediate condition in terms of binder fatigue tolerance. Based on this analysis, the 2.3% content was adopted for production of asphalt mixtures evaluated in subsequent stages of this study.

#### Storage Stability

The storage stability test was conducted in order to evaluate the susceptibility to phase separation of the nanomodified polymer-modified asphalt binder at the selected content of 2.3% during thermal storage. Table 10 presents the nonrecoverable creep compliance (Jnr_3.2_) and percent recovery (%R_3.2_) results obtained from the MSCR test under a stress level of 3.2 kPa, at temperatures of 70 °C, 76 °C, and 82 °C, for the upper (top) and lower (bottom) portions of the samples subjected to the storage stability procedure.

The reference polymer-modified asphalt binder exhibited marked top-to-bottom rheological differences after thermal storage. The Jnr_3.2_ variation exceeded 250% at all evaluated temperatures, indicating the formation of a pronounced rheological gradient along the binder column. This behavior is consistent with the thermodynamic instability commonly observed in polymer-modified asphalt binders, in which differences in compatibility between phases may promote segregation during storage [93].

The nanomodified binder containing MWCNTs and nano-Al_2_O_3_ showed lower top-to-bottom differences than the reference binder, especially for Jnr_3.2_. The residual Jnr_3.2_ variation remained between 55.8% and 58.6% in absolute value across the three temperatures. The %R_3.2_ results showed a similar trend, suggesting a comparatively more uniform elastic response after thermal storage. These results indicate that the hybrid nanomodification reduced the rheological gradient associated with phase separation.

This behavior may be related to the effect of the nanomaterials on the microstructure of the polymer-modified binder, possibly restricting the relative mobility between phases during thermal storage. Similar trends have been reported for SBS-modified binders containing nano-Al_2_O_3_, in which improved phase compatibility was associated with reinforcement of the binder nanostructure and enhancement of the polymer-binder interface [22]. In the present study, although the individual contribution of each nanomaterial could not be isolated, the combined incorporation of nano-Al_2_O_3_ and MWCNTs appears to have contributed to reducing the top-to-bottom rheological gradient.

However, the residual Jnr_3.2_ difference of 55.8–58.6% shows that phase separation was reduced, but not eliminated. Therefore, the nanomodified binder should be interpreted as more storage-compatible than the reference binder, rather than fully storage-stable. From a practical perspective, this residual gradient may still be relevant under industrial storage and transport conditions, especially during prolonged static storage at high-temperature. Thus, procedures commonly adopted for polymer-modified binders, such as controlled storage temperature, limited storage time, and adequate recirculation or agitation before transport, sampling, and mixture production, remain advisable.

### 3.5. Evaluation of Mechanical Performance and Healing Capacity of Asphalt Mixtures

#### 3.5.1. Susceptibility to Permanent Deformation

**Figure 14:**
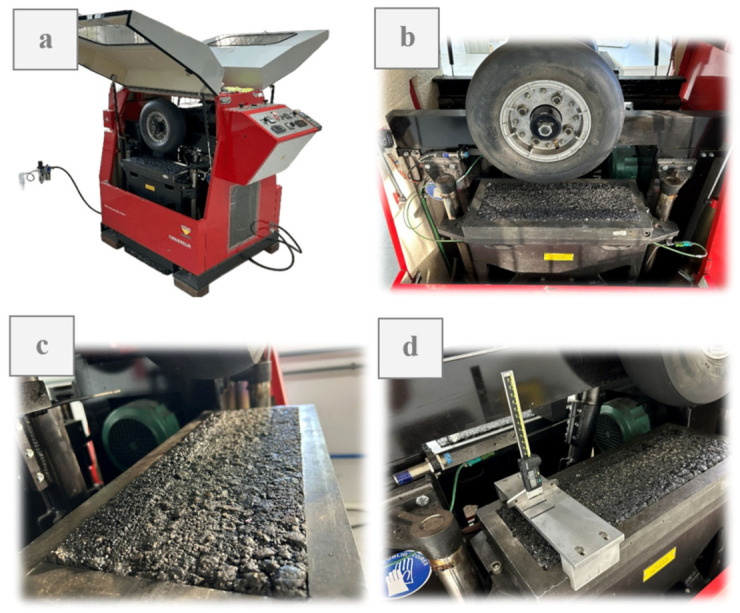
Wheel tracking test using the Orniéreur device: (**a**) general view of the equipment; (**b**) test configuration with loaded wheel; (**c**) asphalt mixture specimen after compaction; (**d**) measurement of rut depth using a digital gauge.

**Figure 15:**
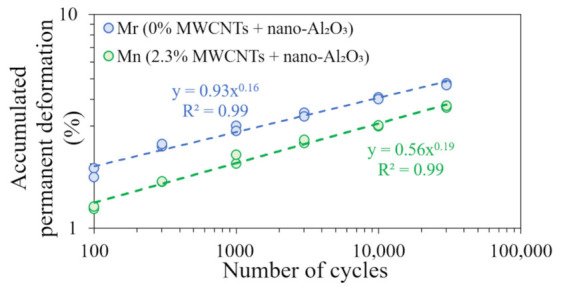
Evolution of rut depth (relative to slab thickness) versus number of loading cycles for reference and nanomodified asphalt mixtures containing 2.3% of MWCNTs + nano-Al_2_O_3_.

Susceptibility to permanent deformation of asphalt mixtures was evaluated using the wheel tracking test with the Orniéreur device (Figure 14). Figure 15 presents the evolution of rut depth relative to slab thickness as a function of the number of applied cycles.

The power regressions of the form y = ax^b^ exhibited high coefficients of determination (R^2^ = 0.99 for both mixtures), indicating good model fitting to rutting progression with increasing number of cycles. Based on the fitted equations, a consistent reduction in rut depth is observed for the nanomodified mixture at all evaluated loading levels. At 300 cycles, the estimated rut depth decreased from 2.3% (reference mixture) to 1.6% (30.5% reduction). At 3000 cycles, the estimated reduction was 26.4% (from 3.4% to 2.5%), while at 30,000 cycles it reached 22.1% (from 4.9% to 3.8%).

The mixture containing 2.3% MWCNTs + nano-Al_2_O_3_ exhibited less strain accumulation throughout the test, indicating higher resistance to permanent deformation under repeated loading. This behavior is consistent with the rheological results obtained for the binders, in which nanomodification promoted increased viscosity, higher high-temperature performance grade (PGH), reduced nonrecoverable creep compliance (Jnr_3.2_), and increased percent recovery (%R_3.2_). Studies on CNTs in nanomodified mixtures and binders report similar behavior, associating this improvement with increased stiffness and greater resistance to accumulation of permanent deformation [13,88].

#### 3.5.2. Fatigue Resistance

**Figure 16:**
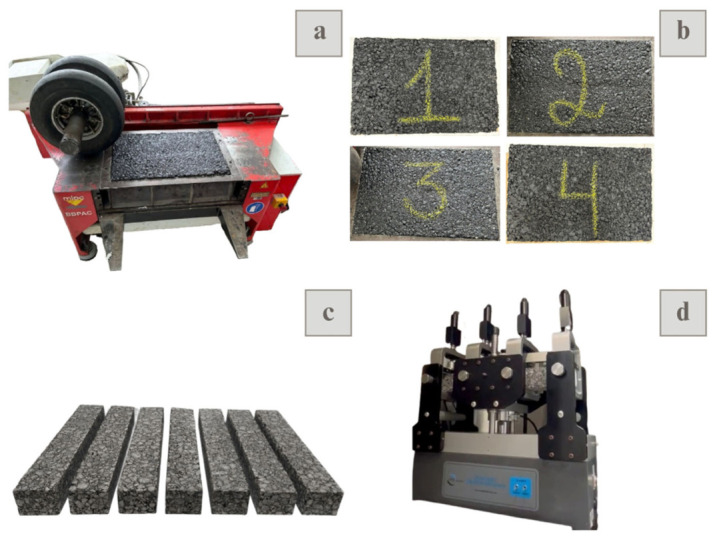
Four-point bending fatigue test: (**a**) compaction of asphalt mixture slabs (60 × 40 × 9 cm) using a French compactor BBPAC (MLPC^®^), developed by VECTRA (currently NextRoad, Fontaine-lès-Dijon, France); (**b**) nanomodified slabs after compaction; (**c**) specimens (38.1 × 6.35 × 5.08 cm) obtained by cutting; (**d**) test using a four-point bending device (IPC Global, Boronia, Australia).

**Figure 17:**
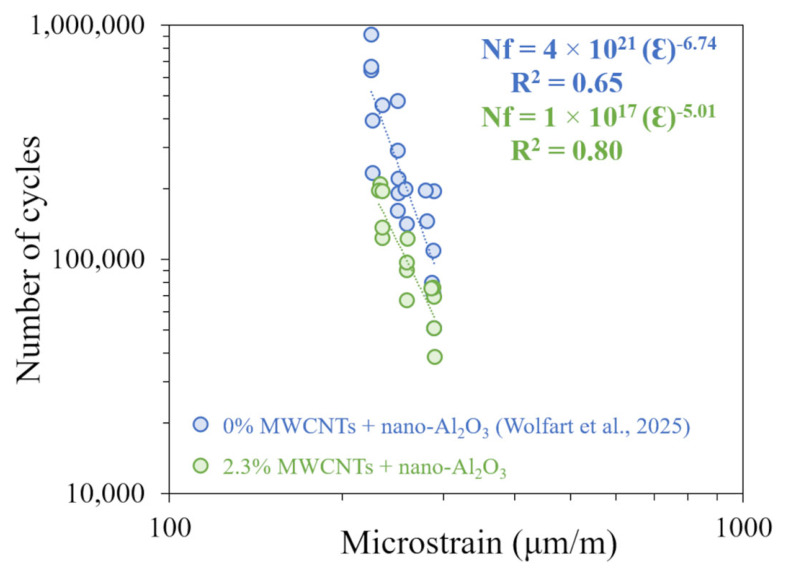
Fatigue curves (number of cycles versus microstrain) obtained in the four-point bending test for the reference mixture [18] and the mixture containing 2.3% MWCNTs + nano-Al_2_O_3_ (20 °C, 10 Hz).

Fatigue resistance of the mixtures was evaluated using the four-point bending test (20 °C and 10 Hz), as shown in Figure 16. Figure 17 presents the fatigue data for the reference mixture, obtained from Wolfart et al. [18], and for the nanomodified mixture containing 2.3% MWCNTs + nano-Al_2_O_3_. Both datasets were fitted using the power-law model N = a(*ε*)^b^.

Parameter a (Figure 17), associated with the magnitude of fatigue life, showed a significant reduction with the incorporation of nanomaterials, decreasing from 4 × 10^21^ to 1 × 10^17^. This result indicates a reduction in the ability of the mixture to resist fatigue damage under cyclic loading. The exponent b (Figure 17) decreased in magnitude, from −6.74 to −5.01, indicating lower sensitivity of fatigue life to variations in strain.

These changes are reflected in the fatigue curves (Figure 17), in which the nanomodified mixture presents a lower number of cycles to failure over the entire range of strain levels analyzed. For 250 μm/m, the reduction was from 255,894 to 118,401 cycles, corresponding to 53.7%. The Mixture Fatigue Factor (FFM) followed this trend, decreasing from 2.70 to 2.43, confirming the overall reduction in fatigue damage tolerance of the nanomodified mixture.

The reduction in fatigue life observed for the nanomodified mixture can be interpreted as a consequence of the increase in stiffness promoted by the incorporation of nanomaterials. Although this stiffening effect was beneficial for resistance to permanent deformation, it also made the material less tolerant to damage accumulation and accommodation under cyclic loading. Under strain-controlled conditions, this combination of higher stiffness and increased brittleness tends to reduce fatigue life, especially at lower strain amplitudes, where the reference mixture showed a higher capacity to withstand repeated loading. This behavior is consistent with the literature, which recognizes excessive stiffness as a potentially detrimental factor for fatigue performance under this type of loading condition [94].

#### 3.5.3. Healing Capacity

Healing capacity of the asphalt mixtures (Mr and Mn) was evaluated by comparing the results obtained before and after application of the healing protocol, as described in Section 2.7, using fatigue life healing and dynamic modulus healing indices (FHI and DMHI). In addition, normalized healing efficiency was determined for each mixture.

Table 11 presents the absolute fatigue results and the Fatigue Healing Index (FHI), stratified by strain level. The values for the reference mixture (Mr) were obtained from Wolfart et al. [18].

As observed in Section 3.5.2, the nanomodified mixture (Mn) exhibited a lower initial fatigue life than the reference mixture (Mr). However, it showed greater consistency in healing capacity, maintaining FHI values between 23% and 28% for all analyzed strain amplitudes, whereas the reference mixture showed a reduction from 22% to 6% with decreasing strain.

In addition, the nanomodified mixture exhibited lower variability in the original fatigue-life results, as indicated by the coefficients of variation. Overall, the results indicate that the incorporation of MWCNTs and nano-Al_2_O_3_ contributes to improved healing efficiency, reducing the sensitivity of healing performance to the applied strain amplitude.

**Figure 18:**
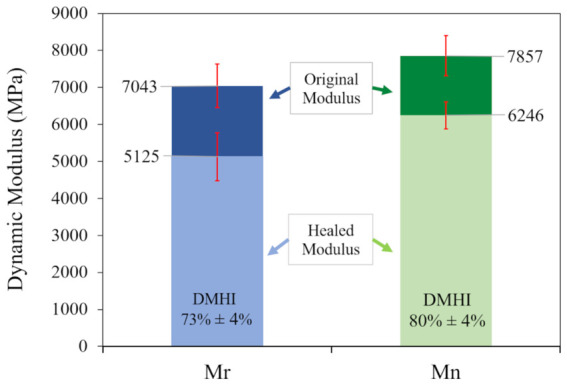
Dynamic modulus healing index (DMHI) of the reference mixture (Mr) [50] and the nanomodified mixture containing 2.3% MWCNTs + nano-Al_2_O_3_ (Mn).

Figure 18 presents the Dynamic Modulus Healing Index (DMHI) for the reference mixture (Mr) and the nanomodified mixture containing 2.3% MWCNTs + nano-Al_2_O_3_ (Mn). The “original” values correspond to the initial dynamic modulus at the 100th cycle of the first fatigue stage, while the “healed” values correspond to the initial dynamic modulus at the 100th cycle of the second stage, after microwave heating.

The nanomodified mixture exhibited a higher original dynamic modulus (7857 MPa) compared to the reference mixture (7043 MPa), as well as a higher value after healing (6246 MPa versus 5125 MPa). As a result, the DMHI increased from 73% (±4%) to 80% (±4%).

This result indicates a greater capacity for stiffness recovery after fatigue damage in the nanomodified mixture. This behavior may be associated with improved thermal efficiency of the material during microwave heating, as indicated by the thermal conductivity results presented in Section 3.3.

The simultaneous improvement in FHI and DMHI, despite the lower initial fatigue resistance of the nanomodified mixture, indicates that fatigue damage tolerance under cyclic loading and thermal healing capacity are not necessarily directly related properties. In the present study, nanomodification reduced fatigue life of the mixture under strain-controlled conditions, but improved its response during the heating and resting cycle adopted in the healing protocol. Thus, the results suggest that the nanomodified mixture was less resistant to initial damage but more responsive to recovery after thermal induction by microwave heating [5,13,14,20].

Considering all analyzed strain amplitudes (227–288 μm/m), the nanomodified mixture exhibited a mean FHI of 25.1 ± 6.7%, compared with 15.5 ± 9.0% for the reference mixture. This difference corresponds to a relative increase of 61.9% in fatigue-life recovery.

The practical balance between the fatigue penalty and the healing gain depends on the service condition. The 53.7% reduction was measured under strain-controlled loading at fixed amplitudes, whereas in service the higher stiffness of the nanomodified mixture tends to reduce the strain level in the asphalt layer [95]. In addition, the FHI of the nanomodified mixture remained between 23% and 28% at all amplitudes, while that of the reference mixture dropped to 6% at the lowest strain. The benefit is therefore expected to be more pronounced at low strain levels, typical of robust structures under heavy traffic, and could be further exploited in maintenance strategies based on periodic induced heating.

Based on these results, healing normalized by the energy density applied during microwave heating was determined. Considering the microwave power of 900 W and an exposure time of 140 s, the total energy supplied was 126,000 J. The calculated energy density was approximately 1.01 × 10^8^ J/m^3^ for both mixtures.

Normalization by the applied energy density yielded healing-efficiency values of 15.4 × 10^−8^ (J/m^3^)^−1^ for the reference mixture and 24.8 × 10^−8^ (J/m^3^)^−1^ for the nanomodified mixture. These results indicate that, for the same order of magnitude of applied energy, the nanomodified mixture presents higher healing efficiency.

Thus, the advantage of the hybrid system does not appear to be associated only with higher achieved temperatures, but also with improved healing performance under the same order of magnitude of supplied energy. This interpretation should be understood in terms of comparative efficiency of the process, and not as a direct measure of the energy effectively absorbed by each mixture. Studies with conductive agents in asphalt mixtures report similar trends, with increased healing as internal heat conduction networks become more efficient [13,14,20].

#### 3.5.4. Thermal Response of Asphalt Mixtures Under Microwave Heating

**Figure 19:**
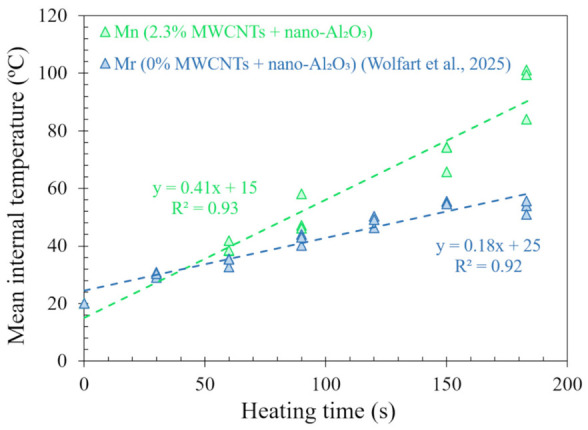
Mean internal temperature as a function of microwave heating time for reference [18] and nanomodified asphalt mixtures containing 2.3% MWCNTs + nano-Al_2_O_3_, including linear regression fitting.

Thermal response of asphalt mixtures was evaluated based on the determination of mean internal temperature after different microwave heating times. The values for the reference mixture were adopted from Wolfart et al. [18], while the temperature of the nanomodified mixture was experimentally determined in this study. Figure 19 presents the evolution of mean internal temperature as a function of heating time for both mixtures.

An approximately linear increase in temperature is observed in Figure 19, with high coefficients of determination (R^2^ = 0.92 for the reference mixture and R^2^ = 0.93 for the nanomodified mixture), indicating good consistency of the experimental data.

The nanomodified mixture exhibited a mean heating rate of 0.41 °C/s, compared with 0.18 °C/s for the reference mixture, corresponding to an increase of approximately 127.8%. After 180 s of heating, the nanomodified mixture reached 88.8 °C, whereas the reference mixture reached 57.4 °C.

The more pronounced thermal response of the nanomodified mixture may be associated with the presence of MWCNTs and nano-Al_2_O_3_, which together may have enhanced both interaction with the electromagnetic field and internal heat redistribution. Regardless of the predominant mechanism, the results show that the nanomodified mixture heated more rapidly and reached higher internal temperatures than the reference mixture. This aspect is relevant because healing depends not only on higher temperatures, but also on sufficiently rapid and distributed heating to increase binder mobility in damaged regions. In this sense, the results suggest that the improved thermal response of the nanocomposite contributed to the enhanced healing performance observed.

## 4. Conclusions

This study investigated the combined incorporation of MWCNTs and nano-Al_2_O_3_ into an SBS-modified asphalt binder and evaluated its effects on thermal conductivity, rheological response, rutting susceptibility, fatigue resistance, and microwave-induced healing efficiency. The following conclusions were drawn from the experimental results:

- The nanomodified binder exhibited a smaller top-to-bottom rheological gradient than the reference binder, although a Jnr_3.2_ difference of approximately 55.8–58.6% remained after thermal storage;
- Hybrid nanomodification improved the high-temperature performance of both the binder and asphalt mixture, as demonstrated by lower Jnr_3.2_, higher %R_3.2_, and a 22.1% reduction in rut depth after 30,000 wheel-tracking cycles;
- Nanomodification reduced fatigue life under strain-controlled loading. Within the investigated range, the number of cycles to failure at 250 μm/m decreased by 53.7%, representing a clear trade-off against the improvements in rutting resistance and healing performance;
- Incorporation of 2.3 wt.% MWCNTs + nano-Al_2_O_3_ increased binder thermal conductivity by 24.2% and the mixture heating rate by 127.8%, from 0.18 to 0.41 °C/s. This enhancement was the principal effect targeted in the study;
- The enhanced thermal response resulted in greater microwave-induced healing. The Dynamic Modulus Healing Index increased from 73% to 80%, and the mean Fatigue Healing Index increased from 15.5% to 25.1% over the strain range of 227–288 μm/m. The latter result corresponds to a relative increase of 61.9% in fatigue-life recovery under the same order of magnitude of supplied energy;

Overall, incorporating 2.3 wt.% MWCNTs + nano-Al_2_O_3_ improved resistance to permanent deformation and microwave-induced healing performance, although it reduced fatigue tolerance under strain-controlled loading. The improvement in healing appears to be associated with the higher thermal conductivity of the nanomodified binder and the more pronounced thermal response of the asphalt mixture during microwave heating. Hybrid nanomodification with MWCNTs and nano-Al_2_O_3_ therefore shows potential for the development of asphalt mixtures with multifunctional behavior, particularly for maintenance strategies involving induced heating.

The following directions are recommended for further work:

- Healing should be evaluated at damage levels other than the 50% reduction in the initial dynamic modulus adopted here, such as reductions of 30% or 40%, under different rest periods and rest temperatures, and over successive damage and healing cycles, so that the persistence of the reported gain after repeated activation can be verified;
- The fatigue response should be assessed at the structural scale through mechanistic analyses;
- Asymmetric MWCNT to nano-Al_2_O_3_ ratios and lower contents should be investigated;
- Complementary techniques, such as SEM or TEM imaging, Raman spectroscopy, and quantitative dispersion analysis, should be used to investigate the microstructural mechanisms proposed in this study.

## Figures and Tables

**Table 1:** Physical properties of the SBS-modified asphalt binder before and after short-term aging in the rolling thin-film oven test (RTFOT) [26].

Properties	Method	Unaged	RTFOT
Softening point (°C)	NBR 6560 [27]	74.4	—
Softening point variation (°C)	NBR 6560 [27]	—	3.4
Penetration, 100 g, 5 s, 25 °C (0.1 mm)	NBR 6576 [28]	49	—
Retained penetration, 25 °C (%)	NBR 6576 [28]	—	69.1
Elastic recovery, 20 cm, 25 °C (%)	NBR 15086 [29]	88	89.7
Flash point (°C)	NBR 11341 [30]	290	—
Solubility in trichloroethylene (% by mass)	NBR 14855 [31]	99.9	—
Specific gravity at 25 °C (kg/m^3^)	NBR 6296 [32]	1.011	—
Mass change RTFOT (%)	T 240 [33]	—	0.3

**Table 2:** Physical and morphological properties of the nanomaterials [22,34].

Property	Nano-Al_2_O_3_	MWCNTs
Mean particle size (nm)	20	—
External diameter (nm)	—	50–80
Internal diameter (nm)	—	5–15
Mean length (µm))	—	10–20
Density (g/cm^3^)	3.5–3.9	2.1
Specific surface area (m^2^/g)	100	60–80

**Table 3:** Physical and mechanical properties of the mineral aggregates [36].

Test	Standard	Result
Los Angeles abrasion	C131 [37]	18.64%
Coarse aggregate absorption	C127 [38]	1.43%
Coarse aggregate angularity	D5821 [39]	100%
Fine aggregate angularity	C1252 [40]	52.36%
Sand equivalent	T 176 [41]	71.95%
Particle shape index of coarse aggregate	NBR 6954 [42]	Cubic
Bulk specific gravity of coarse aggregate	C127 [38]	2.592 g/cm^3^
Real specific gravity of coarse aggregate	C127 [38]	2.648 g/cm^3^
Real specific gravity of fine aggregate	ME 084 [43]	2.674 g/cm^3^
Real specific gravity of filler	ME 085 [44]	2.803 g/cm^3^
Deleterious material	T 112 [45]	0.00%
Soundness	C 88 [46]	1.59%

**Table 4:** Aggregate gradation used in the asphalt mixture [34].

Sieve—ASTM Series	Opening Size (mm)	Passing Material (%)
3/4″	19.00	100
1/2″	12.70	77.5
3/8″	9.51	61.3
No. 4	4.76	43.3
No. 10	2.00	24.3
No. 16	1.19	17.4
No. 30	0.595	12.6
No. 50	0.297	9.8
No. 100	0.149	7.6
No. 200	0.074	5.4

**Table 5:** Asphalt nanocomposites produced with different combined contents of MWCNTs and nano-Al_2_O_3_.

Sample (S)	MWCNTs (wt.%)	Nano-Al_2_O_3_ (wt.%)
S0 (reference)	0%	0%
S1	0.5%	0.5%
S2	1%	1%
S3	1.5%	1.5%
S4	2%	2%
S6	3%	3%

**Table 6:** Rheological experimental program for the asphalt nanocomposites.

Test	Sample (S)	Number of Samples	Aging Condition	Standard
Apparent viscosity	S0–S6	1	Unaged	ASTM D4402/D 4402M [55]
PGH (High-temperature)	S0–S6	2	Unaged and RTFOT	ASTM D7175/D6373 [54,56]
MSCR	S0–S6	2	RTFOT	ASTM D7405 [61]
LAS	S0–S6	4	RTFOT	AASHTO T 391-20 [58]

**Table 7:** Experimental program used to evaluate the mechanical performance and healing capacity of the asphalt mixtures. n: number of specimens tested.

Test	n (Mr)	n (Mn)	Specimen Dimension (cm)	Standard/Procedure
Permanent deformation	2	2	60 × 40 × 9	EN 12697-22 [66]
Fatigue	20 *	14	38.1 × 6.35 × 5.08	EN 12697-24 [67]
Healing	20 *	14	38.1 × 6.35 × 5.08	-
Heating	19 *	13	38.1 × 6.35 × 5.08	-

* Data for the reference mixture were obtained from Wolfart et al. [18]. That study was conducted by the same research group using the same laboratory, equipment, procedures, aggregate gradation, mineral aggregates, reference SBS-modified binder, and volumetric parameters. However, the experiments were performed in a separate experimental campaign, which should be considered when interpreting the comparisons.

**Table 8:** Percentage mass change in asphalt nanocomposite after short-term aging (RTFOT).

Sample (S)	Mass Change (%)	Standard Deviation (%)
S0 (reference)	0.34%	0.01%
S1	0.31%	0.01%
S2	0.31%	0.01%
S3	0.39%	0.00%
S4	0.40%	0.01%
S6	0.40%	0.00%

**Table 9:** Rheological and thermal properties of asphalt nanocomposites. ^(a)^ values obtained from regression models; ^(b)^ experimental values; ^(c)^ values obtained by interpolation between experimental data of S2 and S3.

Parameter	S0	S1	S2	S2.3	S3	S4	S6
Viscosity 135 °C (cP) ^(a)^	1982	2411	2854	2992	3311	3782	4766
Viscosity 150 °C (cP) ^(a)^	945	1078	1235	1287	1416	1621	2103
Viscosity 177 °C (cP) ^(a)^	329	366	415	432	476	549	731
PGH Unaged (°C) ^(a)^	82.33	84.07	85.69	86.15	87.19	88.57	90.97
	82-XX	82-XX	82-XX	82-XX	82-XX	88-XX	88-XX
PGH after RTFOT (°C) ^(a)^	82.71	83.42	84.15	84.37	84.9	85.67	87.27
	82-XX	82-XX	82-XX	82-XX	82-XX	82-XX	82-XX
Aging Index—AI 58 °C ^(b)(c)^	2.40 ^(b)^	2.11 ^(b)^	1.92 ^(b)^	1.98 ^(c)^	2.11 ^(b)^	2.33 ^(b)^	1.97 ^(b)^
Aging Index—AI 64 °C ^(b)(c)^	2.42 ^(b)^	2.10 ^(b)^	1.92 ^(b)^	1.97 ^(c)^	2.09 ^(b)^	2.29 ^(b)^	1.93 ^(b)^
Aging Index—AI 70 °C ^(b)(c)^	2.45 ^(b)^	2.09 ^(b)^	1.91 ^(b)^	1.95 ^(c)^	2.05 ^(b)^	2.23 ^(b)^	1.89 ^(b)^
Aging Index—AI 76 °C ^(b)(c)^	2.44 ^(b)^	2.04 ^(b)^	1.87 ^(b)^	1.91 ^(c)^	1.99 ^(b)^	2.14 ^(b)^	1.81 ^(b)^
Aging Index—AI 82 °C ^(b)(c)^	2.39 ^(b)^	1.96 ^(b)^	1.82 ^(b)^	1.84 ^(c)^	1.90 ^(b)^	2.04 ^(b)^	1.71 ^(b)^
Aging Index—AI 88 °C ^(b)(c)^	2.30 ^(b)^	1.87 ^(b)^	1.75 ^(b)^	1.76 ^(c)^	1.78 ^(b)^	1.91 ^(b)^	1.60 ^(b)^
Jnr_3.2_ 82 °C (kPa^−1^) ^(a)^	2.71	2.35	2.05	1.97	1.81	1.63	1.45
Jnr_3.2_ 76 °C (kPa^−1^) ^(a)^	0.72	0.67	0.62	0.61	0.57	0.52	0.42
Jnr_3.2_ 70 °C (kPa^−1^) ^(a)^	0.24	0.22	0.20	0.19	0.18	0.16	0.12
%R_3.2_ 82 °C (%) ^(a)^	19.79	21.93	23.69	24.14	25.07	26.07	26.93
%R_3.2_ 76 °C (%) ^(a)^	46.88	49.26	51.3	51.85	53	54.36	56.06
%R_3.2_ 70 °C (%) ^(a)^	67.16	69.34	71.28	71.82	72.98	74.44	76.64
Thermal conductivity (W/m·K) ^(a)^	0.19	0.21	0.23	0.24	0.25	0.27	0.31

**Table 10:** MSCR rheological parameters (Jnr_3.2_ and %R3.2) of the upper (top) and lower (bottom) portions of the samples after the storage stability test.

Sample	Portion	70 °C		76 °C		82 °C	
		%R_3.2_	Jnr_3.2_	%R_3.2_	Jnr_3.2_	%R_3.2_	Jnr_3.2_
0% MWCNTs + nano-Al_2_O_3_ (S0)	Top	68.02%	0.32 kPa^−1^	39.01%	1.12 kPa^−1^	14.31%	3.40 kPa^−1^
	Bottom	53.33%	1.26 kPa^−1^	23.92%	4.24 kPa^−1^	4.35%	12.10 kPa^−1^
	Variation	−21.6%	293.1%	−38.7%	278.6%	−69.6%	255.9%
2.3% MWCNTs + nano-Al_2_O_3_	Top	65.74%	0.60 kPa^−1^	34.80%	2.11 kPa^−1^	9.51%	6.63 kPa^−1^
	Bottom	71.85%	0.26 kPa^−1^	42.35%	0.93 kPa^−1^	16.06%	2.74 kPa^−1^
	Variation	9.29%	−57.6%	21.7%	−55.8%	68.9%	−58.6%

**Table 11:** Mean fatigue life results and Fatigue Healing Index (FHI) under different strain amplitudes. Mr: reference mixture; Mn: nanomodified mixture; CV%: coefficient of variation; SD: standard deviation.

Mixture	Microstrain (μm/m)	Mean Original Cycles (CV%)	Mean Post Healing Cycles (CV%)	Mean FHI (SD)
Mr [18]	286	121,341 (49%)	26,485 (49%)	22% (±4%)
	253	240,299 (48%)	41,616 (57%)	17% (±9%)
	227	550,313 (44%)	25,250 (46%)	6% (±3%)
Mn	288	61,887 (27%)	17,692 (35%)	28% (±3%)
	259	94,146 (24%)	23,786 (62%)	24% (±9%)
	234	172,514 (23%)	40,686 (46%)	23% (±7%)

## Data Availability

The original contributions presented in this study are included in the article. Further inquiries can be directed to the corresponding author.
